# Response to: In-hospital cardiac arrest: evidence and specificities of perioperative cardiac arrest

**DOI:** 10.1186/s13054-023-04314-y

**Published:** 2023-01-17

**Authors:** James Penketh, Jerry P. Nolan

**Affiliations:** 1 Intensive Care Unit, Royal United Hospital, Bath, UK; 2 Warwick Clinical Trials Unit, University of Warwick, Coventry, UK

## Dear Editor,

We thank De Roux et al. for their interest and comments on our review of in-hospital cardiac arrest [1]. We agree completely that perioperative cardiac arrest has many specific features that make it very different from the circumstances of other causes of in-hospital cardiac arrest. Perioperative cardiac arrests are excluded from some in-hospital cardiac arrest studies for this reason. The epidemiology, treatment and outcome from perioperative cardiac arrest warrant a separate review—we certainly did not have enough space within our review to do justice to the topic. The clinical practice recommendations made by the Perioperative Cardiac Arrest (PERIOPCA) Consortium are potentially helpful [2], as are guidelines published previously by another international group [3, 4]. Unfortunately, it is unlikely that we will ever have high-certainty evidence to inform clinical guidelines on the treatment of perioperative cardiac arrest. Best practice guidance inevitably varies depending on which expert group is providing the advice. Currently, the International Liaison Committee on Resuscitation (ILCOR) has the most comprehensive international representation of any group of resuscitation experts but has yet to review the science and publish treatment recommendations on perioperative cardiac arrest. The Advanced Life Support Task Force of ILCOR may review this topic in the future and may be informed partly by the findings of the Royal College of Anaesthetists 7th National Audit Project (NAP7) [5]. The NAP7 steering group has reviewed all perioperative cardiac arrests occurring in UK hospitals over a 1-year period and will report its findings in a series of papers in 2023.

## Data Availability

Not applicable.
